# Cryptic fungal infections: the hidden agenda of plant pathogens

**DOI:** 10.3389/fpls.2014.00506

**Published:** 2014-09-26

**Authors:** Ioannis Stergiopoulos, Thomas R. Gordon

**Affiliations:** Department of Plant Pathology, University of California DavisDavis, CA, USA

**Keywords:** fungi, symbiosis, pathogenesis, virulence, disease, cryptic infection, plant

## Introduction

Host-microbe interactions have traditionally been viewed primarily from the perspective of pathogenesis and disease. The principal assumption is that a susceptible host will support microbial growth inside host tissues, leading eventually to the development of symptoms and disease. Inversely, non-susceptible (resistant) hosts will block pathogen infection and the expression of disease (Casadevall and Pirofski, 2001; Hok et al., 2010). Compatible host-microbe interactions, however, do not always result in overt negative effects, and asymptomatic or cryptic fungal infections are increasingly recognized as a common feature of many symbiotic associations between fungi and their hosts (Rodriguez et al., 2009; Porras-Alfaro and Bayman, 2011; Malcolm et al., 2013). It is now generally accepted that extensive intra (Stone, 1987; Bernardi-Wenzel et al., 2010) and intercellular (Schulz and Boyle, 2006) (Gómez-Vidal et al., 2006; Rodriguez et al., 2009) colonization of plants by endophytic fungi and other microorganisms (Thomas and Sekhar, 2014) is the norm, and that it would not be unusual for an individual host to sustain the growth and development of dozens of microbial taxa (Suryanarayanan et al., 2003; Ganley and Newcombe, 2006; Gazis and Chaverri, 2010; De Siqueira et al., 2011). Consequently, it should also not come as a surprise to discover that many of the fungi that we know only as pathogens have in fact a much broader repertoire of ecological interactions with their hosts, which may include the capacity to infect and colonize them without inducing any visible damage (Malcolm et al., 2013). Despite this fact, we still largely regard these relationships as aberrations and not typical of the manner in which fungi interact with their hosts, often excluding them from the study of host-microbe interactions with potentially adverse consequences on disease control (Filipe et al., 2012). Here we use selected examples to emphasize how cryptic associations between fungi and plants can influence our capacity to manage plant diseases, while also providing insights into the origins of plant pathogens.

## Tiptoeing on the symbiosis balance beam

Fungi can participate in a diverse array of intimate symbiotic relationships with their hosts, ranging from harmful to beneficial (Newton et al., 2010). Broadly speaking, symbiotic relationships are categorized as parasitic, commensal or mutualistic. In parasitism, the parasite benefits from the association while the host is harmed. In commensalism, one partner benefits from the interaction while the other appears to be unaffected, whereas in mutualism, both partners benefit from the interaction (Casadevall and Pirofski, 2000). Although these categories are useful for conceptualizing relationships between hosts and microbes, they do not reflect the continuum that truly exists or the dynamic nature of the interactions, which can result in a single host-fungus combination displaying features of parasitism, commensalism, and mutualism (Casadevall and Pirofski, 2003; Schulz and Boyle, 2005; Newton et al., 2010). It is thus not uncommon for the balance in a symbiotic interaction to shift depending on environmental conditions, the genetics of the host, the microbial species and the interaction stage (Casadevall and Pirofski, 2003; Kogel et al., 2006; Casadevall, 2007; Giauque and Hawkes, 2013; Iliev and Underhill, 2013). Consequently, fungi may adopt contrasting lifestyles, by completing their life-cycle as pathogens on some hosts, while living as commensals or mutualists on others (Schulz and Boyle, 2005; Malcolm et al., 2013). Disease in that perspective could be perceived as an unbalanced equilibrium between *“supply-and-demand”* in host-microbe interactions and a breakdown in the balanced and potentially (mutually) beneficial co-existence between hosts and microbes (Casadevall and Pirofski, 2000; Newton et al., 2010).

## Hidden in plain sight

Asymptomatic or cryptic plant infections by commensal or mutualistic fungi have been traditionally associated with endophytes (Rodriguez et al., 2009; Porras-Alfaro and Bayman, 2011). The term was first coined in the mid-19th century by Anton de Bary (1879), the founding father of modern-day plant pathology, and was loosely translated as *“any fungus or bacterium found inside plant tissues”* (Bary, 1879). Today, however, endophytes are often categorized as “fungi or bacteria that invade tissues of living plants and cause no apparent effect” (Wilson, 1995). Of course this definition specifically excludes microorganisms having a conspicuous beneficial effect, such as mycorrhizae and nodule-forming bacteria that maintain mutualistic associations with their hosts. It also excludes microorganisms with conspicuous negative effects, with the exception that pathogens may be included to the extent that they have an endophytic (or latent) stage (Wilson, 1995; Porras-Alfaro and Bayman, 2011). Almost every vascular plant species examined to date has been found to harbor endophytic fungi. Consequently, there exists an enormous diversity both in species as well as symbiotic and ecological functions of endophytic fungi (Arnold, 2007; Arnold and Lutzoni, 2007; Rodriguez et al., 2009). Among the best studied endophytes are the so-called Clavicipitaceous endophytes of the genus *Neotyphodium* (teleomorph = *Epichloë*) and related genera, which form obligate biotrophic associations with temperate grasses and play an important role in host physiology by enhancing growth and conferring abiotic and biotic stress tolerance. Due to their agronomic importance, Clavicipitaceous endophytes have been extensively reviewed elsewhere (Saikkonen et al., 1998; Schardl et al., 2004, 2009; Rodriguez et al., 2009), and thus will not be considered further here.

## The secret life of plant pathogens

Many fungi that establish symptomless associations with their hosts, however, are not obligate endophytes but rather may switch between pathogenic and commensal or mutualistic lifestyles, depending on environmental conditions and the host. This is well illustrated by strains of *Fusarium oxysporum*, which are regarded as host-specific because they cause disease on a narrow range of genotypes, but which also infect and grow asymptomatically within the root cortex of crop plants (Gordon et al., 1989). The taxonomic breadth of species that host cryptic infections by *F. oxysporum*, suggests that this mode of existence may be the norm, whereas interactions causing conspicuous damage are an aberration. Consistent with this view is the widespread occurrence of *F. oxysporum* in native plant communities as a benign colonizer of grasses and other plant hosts (Gordon and Martyn, 1997). Where grassland soils have been brought under cultivation, resident populations of *F. oxysporum* may persist (Gordon et al., 1992) and colonize whatever crops are made available to them. Over time, chance combinations of plant and fungal genotypes may result in interactions that are damaging to the host. This mode of origin could explain the emergence of *F. oxysporum* strains that cause wilt diseases on cotton (Wang et al., 2004; Chakrabarti et al., 2011). If such fungi with a potentially pathogenic lifestyle retain their ancestral capacity to exploit other hosts as endophytes, prospects for management through crop rotation will be diminished.

The significance of cryptic infections is not limited to agroecosystems, as may be seen in the example of *Fusarium circinatum*, a pathogen of pines in native forests, but now also known to colonize grass species as an endophyte (Swett and Gordon, 2012). Such grass isolates are virulent on pine and can potentially be dispersed asymptomatically over multiple spatial and temporal scales, thus turning cryptically infected grasses into reservoirs of pathogen inoculum and accelerating disease transmission by facilitating horizontal grass-to-grass movement of the fungus between isolated stands of susceptible pines. Consequently, management strategies that are restricted to consideration of only symptomatic hosts without taking into an account the pathogen's true host-range may not be effective. Acknowledging that plant pathogens can be dispersed in asymptomatic hosts over long distances is also of paramount importance when considering quarantine measures against invasive species such as *F. circinatum*.

## “Avirulent” strains: not such losers after all

Whereas fungi such as *F. circinatum* adopt different symbiotic lifestyles on genetically distant host species, variation can also be found among fungal isolates infecting the same host species, thus challenging the concept of a species having a single symbiotic lifestyle on a particular host (Barrett et al., 2009). Although intraspecific variation in pathogenicity can be a consequence of quantitative host resistance, cases in which endophytes represent seemingly avirulent (non-pathogenic) strains of known pathogenic species (Freeman and Rodriguez, 1993; Redman et al., 1999; Schulz and Boyle, 2006) in systems characterized by qualitative host resistance have been described as well. The hemibiotrophic ascomycete *Zymoseptoria tritici* (formerly known as *Mycosphaerella graminicola*), for example, is a notorious pathogen of wheat, whose interaction with the host conforms to the gene-for-gene relationship (Kema et al., 1996, 2000; Brading et al., 2002; Palmer and Skinner, 2002). Despite the expected race-specificity, host-pathogen incompatibility in this pathosystem does not necessarily restrict growth of the fungus inside the host, which nevertheless remains free of disease symptoms (Ware, 2006). Such isolates are non-pathogenic in the sense that they cannot complete a cycle of asexual reproduction on the host or induce any externally visible damage (i.e., necrosis and chlorosis), but they may still grow substantially inside plant leaves and engage in sexual recombination with virulent isolates colonizing the same host (Ware, 2006). Hence, their genes are not quickly removed from the gene pool, which consequently affects the disease transmission dynamics and the development of disease epidemics by maintaining virulence heterogeneity in the pathogen population. This could potentially moderate pathogen virulence at a population level and balance the overall cost of virulence, thus overcoming the risk of driving the host to extinction. For host-specific pathogens such as *Z. tritici*, which is known to have co-evolved as a specialized pathogen of wheat, the ability of some isolates to multiply asymptomatically inside the host as non-destructive colonists could reflect an evolutionary adaptation at a population level that maintains allelic diversity at virulence-associated loci, thereby enhancing the prospects for long-term reproductive success of the pathogen (Vanbaalen and Sabelis, 1995; Frank, 1996; Read and Taylor, 2001; Rauch et al., 2003). Furthermore, retaining virulence heterogeneity at a population level could facilitate pathogen transmission by retaining a proportion of the host population alive for some time, thus prolonging the infectious period and enabling a higher number of sexual cycles and consequently ascospores to be produced within a growing season. Ascospores of *Z. tritici*, in particular, are wind transmitted and have been reported to travel for hundreds of kilometers, in contrast to the rain-splash, locally dispersed asexual pycnidiospores. Thus, maintaining a parasitic continuum in a pathogen population between non-destructive endophytic colonizers and virulent isolates may reflect an evolutionary adaptation that increases the long-term reproductive success of the organism. More importantly, the ability of some isolates to grow asymptomatically inside the host in a cryptic or latent manner could offer support to the idea that modern plant–pathogen interactions have arisen from the breakdown of past mutualistic interactions and a switch to a pathogenic lifestyle (Stukenbrock and Mcdonald, 2008; Eaton et al., 2010; Kiers et al., 2010).

## Concluding remarks

The majority of studies in host-microbe interactions have thus far followed a rather reductionist approach, focusing mostly on aspects of pathogenesis and disease. However, it is possible that all of our hard-won knowledge about the activities of some species as pathogens, in fact represents a vanishing small part of the full spectrum of ecological activities that constitute their life history. Thus, for a holistic understanding of host-microbe interactions, it is vital to understand and appreciate the true breadth and dynamic nature of the parasitic, commensal or mutualistic continuum that exists between microbes and their hosts. Failing to do so may compromise efforts to manage species normally perceived as pathogens, and increase the risk of introducing invasive species into new areas. We consequently stress the importance of determining the extent to which fungi with a potentially pathogenic lifestyle exploit host plants that manifest no symptoms, and incorporating this information into epidemiological models and disease management strategies. Future studies should also focus on identifying the genetic and environmental factors that trigger a transition in the trophic interactions between hosts and parasites, and subsequently the phenotypic state of an infection. By studying in parallel the parasitic, mutualistic, and commensal lifestyles of traditional pathogens, key processes in the host-pathogen interplay and molecular communication can be identified that can be exploited for more sustainable disease control measures. Overall, we argue that a paradigm-shift in the study of host-microbe interactions is currently needed, to acknowledge the full extent of trophic interactions between parasites and hosts.

### Conflict of interest statement

The Associate Editor Benjamin Schwessinger declares that, despite being affiliated to the same institution as Ioannis Stergiopoulos and Thomas R. Gordon, the review process was handled objectively and no conflict of interest exists. The authors declare that the research was conducted in the absence of any commercial or financial relationships that could be construed as a potential conflict of interest.
